# An Inexpensive, Multimodal Simulation Model for Teaching Ultrasound Identification of Soft Tissue Pathology and Regional Anesthesia

**DOI:** 10.7759/cureus.37295

**Published:** 2023-04-08

**Authors:** Skylar DeHaan, Renato Rapada, Cody F Newell, Vance M Rothmeyer, Melissa Myers

**Affiliations:** 1 Emergency Medicine, Brooke Army Medical Center, San Antonio, USA

**Keywords:** fascia iliaca compartment block, soft tissue abscess identification, foreign body on ultrasound, emergency medicine procedural competency, emergency medicine training, ultrasound guided regional anesthesia, skills and simulation training, low-cost high-fidelity task trainers, ultrasound phantom, tissue model

## Abstract

Ultrasound identification of soft tissue pathology is a useful skill for the emergency physician, but it requires practice and familiarity to be effective. Given its rising popularity in the Emergency Department, regional anesthesia is another essential skill that requires practice. Realistic models can help create procedural confidence and accuracy. Since entry-level professional-grade models can be cost-prohibitive, the development of simple and affordable models for teaching is valuable for emergency provider education, especially in resource-limited settings.

Other inexpensive models have been produced and discussed in ultrasound; literature; however, no models have yet been designed for the replication of several different modalities in a single model. We developed and successfully tested a meat phantom model utilizing materials available at a local grocery store that can be quickly assembled in a short amount of time with minimal effort. This low-cost, easy-to-make phantom accurately replicates human tissue and pathology and is ideal for learners to practice several skill sets at once.

## Introduction

Soft tissue injury is a common presenting complaint in the Emergency Department, and retained foreign bodies such as wood can be missed up to 93% of the time on plain films [1]. Retained foreign bodies that are not removed can lead to complications, including inflammation, increased risk of infection, and delayed wound healing, making both identification and treatment on initial presentation essential to preventing complications. Due to the poor sensitivity of X-ray in identifying certain foreign bodies, ultrasound is a readily available and portable tool that allows for easy evaluation of wounds for foreign bodies of all varieties, with sensitivities varying from 72% to 99% for radiolucent objects [2,3]. Given the importance of operator skill in effectively locating foreign bodies within soft tissue, it is important to have access to models for training purposes. 

Ultrasound-guided regional anesthesia is part of the skill set for ED physicians and requires practice and familiarity to develop the psychomotor skills necessary for effective utilization in patient care. The interpretation of images when using this modality is directly related to the provider’s level of training [4]. The use of ultrasound guidance also reduces the risk of local anesthetic systemic toxicity when performing peripheral nerve blocks [5]. Since the ability to interpret ultrasound imaging is associated with the successful placement of anesthetic, realistic models serving as surrogates for patient anatomy can be beneficial to learners in honing their skills in needle placement and hydro-dissection under ultrasound guidance. 

Inexpensive models have been produced and discussed in ultrasound literature with a focus on replicating abscesses, and they have been shown to increase diagnostic accuracy in even inexperienced hands [6,7]. However, there are no current models designed specifically for the replication of several different modalities in a single model. A meat phantom is a simple and affordable model that is easy to implement with minimal preparation required on the part of the educator. Given that even entry-level professional-grade models can be cost-prohibitive, the development of simple and affordable models for teaching is valuable for emergency provider education. 

This article was previously presented as a meeting abstract at the 2021 San Antonio Military Health and Universities Research Forum (SURF) on June 10^th^, 2021. 

## Technical report

This simple model was developed utilizing materials available at a local grocery store that can be quickly assembled in a short amount of time with minimal effort. Additional materials readily available to Emergency Physicians were also used. All required materials are listed in Table 1. 

**Table 1:** Itemized list of required materials

Components of the Multimodal Model:	Required Materials:
Meat phantom	5 lb beef chuck roast ($14.65)
Foreign Body Phantom	Scalpel, 10-blade, curved hemostat, Wood splinter or toothpick, plastic eyes, metal needle, pebble
Abscess model	Aquasonic ultrasound gel, 18g needle, 10cc syringe
Nerve block model	Carving knife, 2 straws, approx 0.5-inch diameter, wooden skewer ($2.00), modeling clay ($3.99)

**Figure 1:**
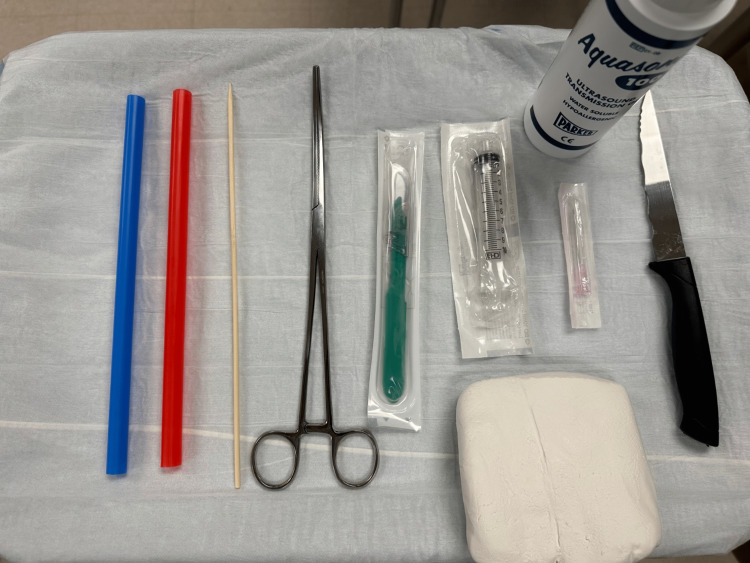
Assembled materials

All materials were obtained before assembly (Figure 1). For the foreign body component of the model, several foreign bodies were inserted at varying depths into the model. The foreign bodies were selected for their variations in shape and representation of materials that are often found embedded in wounds. These objects included a wood splinter, plastic eyes, a metal needle, and a pebble. Each object demonstrates different echogenic properties. The embedding of the objects was accomplished with a 10-blade needle and a curved hemostat, but it can be accomplished with any similar-sized instruments. 

For the abscess portion, 5 mL of Aquasonic ultrasound gel was used. The gel was inserted into the model using an 18g needle just before utilization of the model. This material was chosen due to its similarities to an abscess on imaging and its ease of availability. 

**Figure 2:**
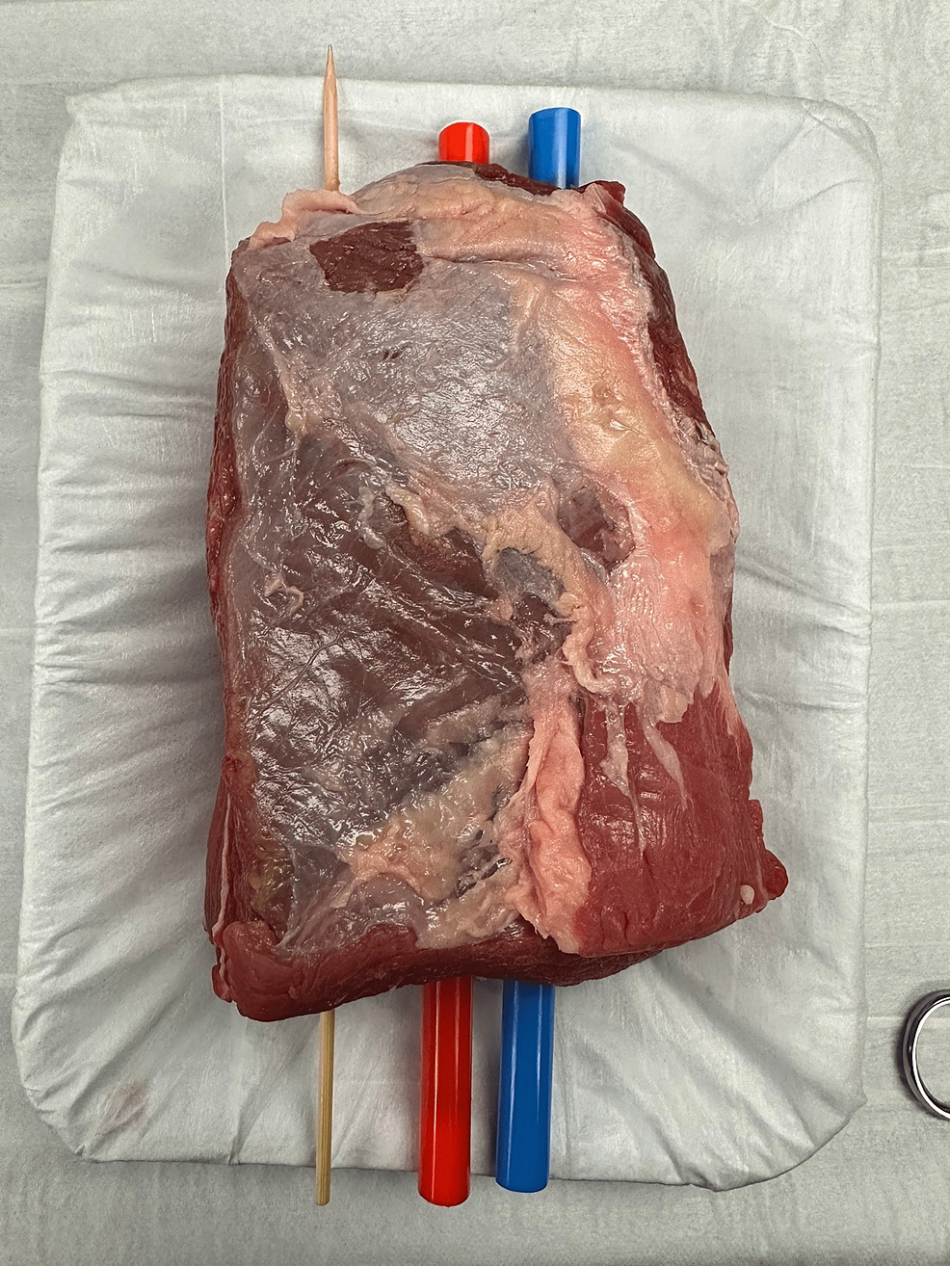
Model after insertion of foreign bodies, ultrasound gel, and simulated nerve bundle

**Figure 3:**
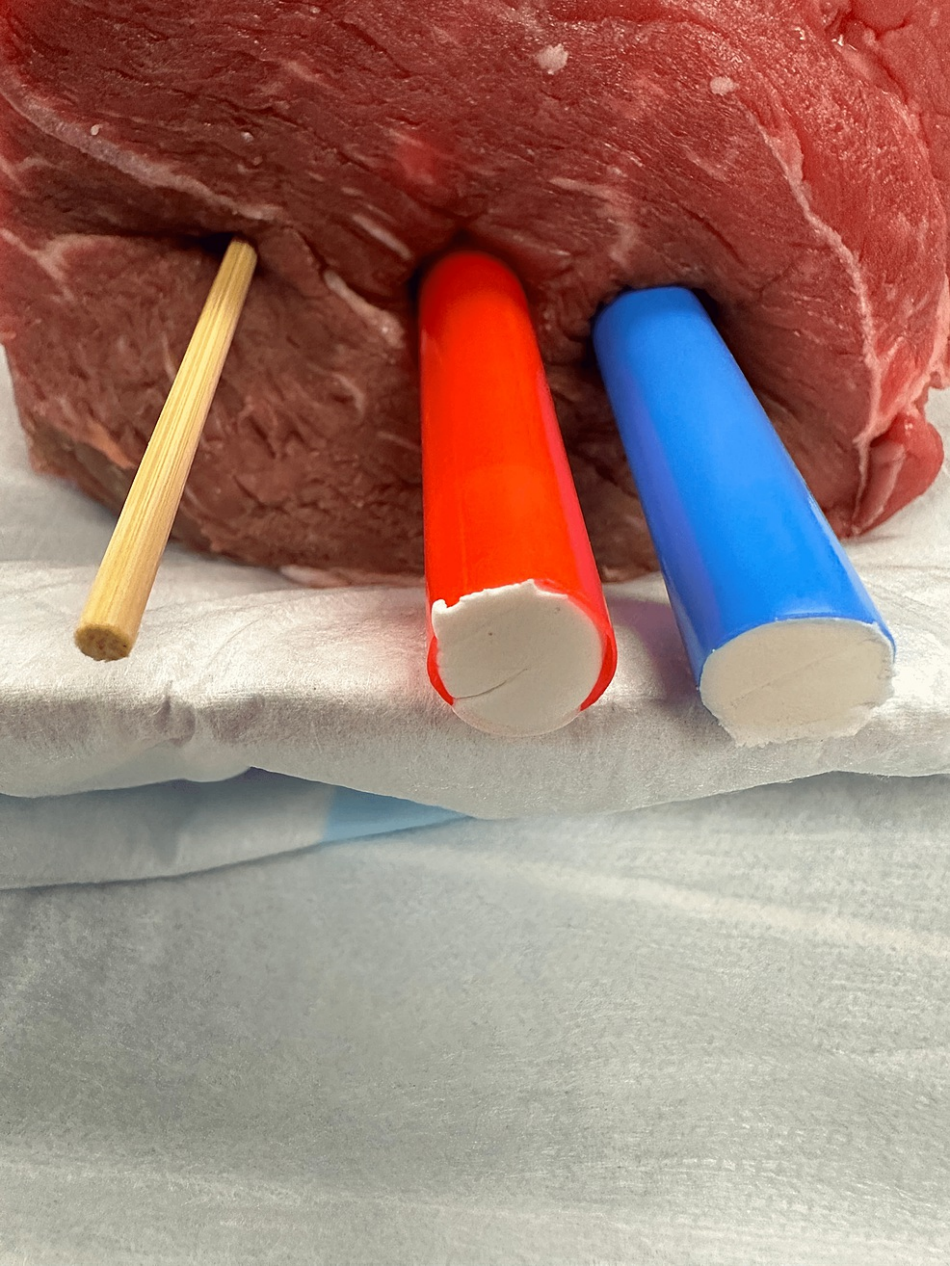
Obtaining a watertight seal using modeling clay after filling straws with water

To create the nerve block component of the model, a standard carving knife was inserted into the middle of the model to create a tract. A red straw, a blue straw, and a wooden skewer were then inserted (Figure 2). Immediately before beginning the class, one end of each straw was blocked using modeling clay. While holding the model upright, tap water was inserted into the straws using a 10cc syringe and an 18-gauge needle. Once the straws were filled with water, the other end of the straws was also capped using modeling clay to create a water-tight seal. (Figure 3). Finally, a thin layer of gel was applied, followed by a layer of plastic wrap to protect learners from directly interacting with raw meat.

The training session was accomplished by having learners evaluate the model using ultrasound to find as much soft tissue pathology as possible without clues on location or the pathology present in the meat phantom. An instructor was located nearby to provide feedback on identification and to notify the learner when all soft tissue pathology was identified. For the regional anesthesia, learners were provided with an 18g spinal needle and a syringe filled with saline to practice in-plane needle guidance and hydro-dissection with the model. A total of 48 participants volunteered to use the models as training aids, and all participants were Emergency Medicine residents of varying training.

**Figure 4:**
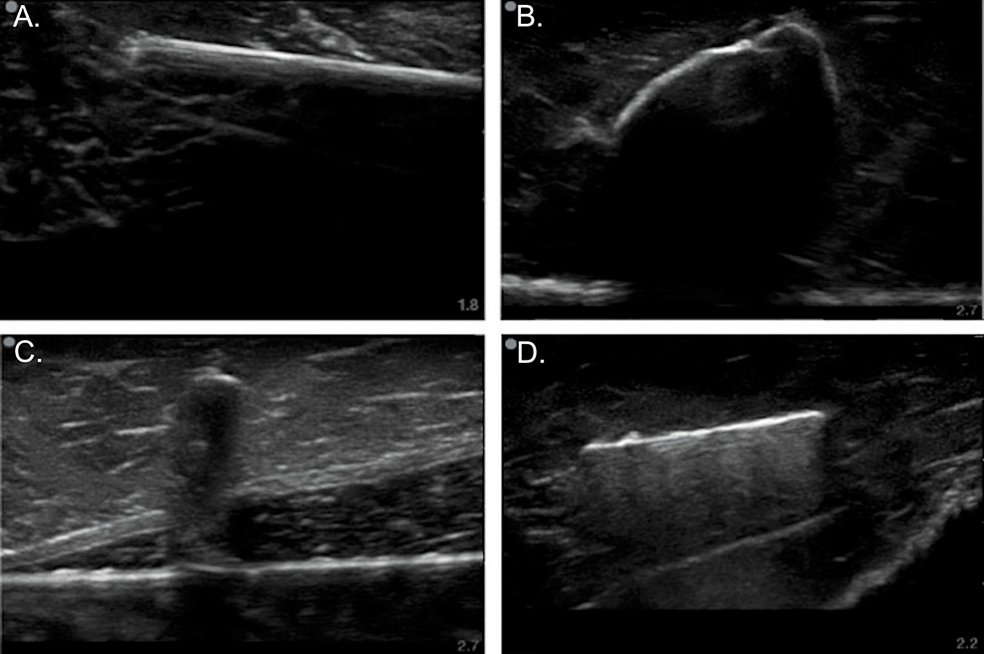
Ultrasound images of foreign bodies Images obtained using the handheld Lumify linear 12- to 4-MHz probe (Philips, Amsterdam, Netherlands)

The foreign bodies varied in their acoustic shadowing based on their properties. Wood (Figure 4A) and pebbles (Figure 4B) showed a clean shadow, while plastic (Figure 4C) showed variable shadowing. Metallic objects showed an expected reverberation artifact (Figure 4D), making them easier to identify. 

**Figure 5:**
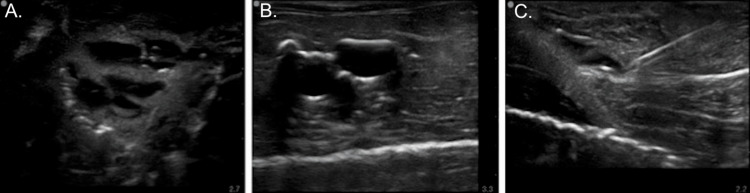
Ultrasound images of an abscess, simulated vessels, and needle guided hydro-dissection Images obtained using the handheld Lumify linear 12- to 4-MHz probe (Philips, Amsterdam, Netherlands)

The ultrasound gel produced ultrasound findings highly consistent with a soft tissue abscess (Figure 5A). 

The fluid-filled straws also provided a realistic impression of the vasculature that is found adjacent to the femoral nerve (Figure 5B). Because the chuck roast has fascia, fascial layers, and muscle tissue similar to that of a live patient, the model gave a realistic appearance on imaging during needle tracking and hydro-dissection (Figure 5C). A post-utilization survey revealed that 94% of participants strongly agreed the meat phantom accurately represented foreign bodies, and 88% of users reported the model aided them in developing the motor skills required to deliver regional anesthetia.

## Discussion

With these inexpensive and realistic meat phantoms, object localization and identification on ultrasound can be realistically simulated and practiced in a low-stakes environment before utilization in a live patient care situation. One significant advantage our model has is the presence of facial planes. Facial planes can mask foreign bodies, as is often encountered in real patients. Similar meat phantom models have shown the overall accuracy of ultrasound in identifying foreign bodies [8]. Given the poor sensitivity of X-ray and the risk of complications when identification of foreign bodies is delayed, developing ultrasound-guided techniques with a simple model can be hugely beneficial to Emergency Providers.

Other publications have also detailed the creation of abscess models, which have been shown to be highly consistent with true soft tissue pathology. A major disadvantage of our model was the dilution of gel into the surrounding tissue, and while our model allowed for abscess identification, using a balloon or other more rigid structure would allow for lancing and needle drainage [9]. This extra step may be time prohibitive to create, but it would be an excellent option for future learners and would help mitigate the disadvantages of our model.

Our model was also able to accurately replicate an artery, vein, and nerve group, specifically the femoral artery, which must be accurately identified when performing regional anesthesia for femoral nerve blocks. As this procedure becomes more commonplace for Emergency Providers, the image interpretation and fine motor skills needed to safely perform it are becoming increasingly important. The presence of fascial layers in the muscle tissue of our model allows for the practice of hydro-dissection of these planes and provides a more realistic learning experience when practicing these skills.

Though other models use a similar material, our model has the distinct advantage over traditional gel trainers in better representing soft tissue. By not including metallic objects to represent the nerve bundle, our model did not have issues with visualization when performing hydro-dissection. While similar results have been achieved by other phantoms, our model uses simple, readily available material that does not require preservation [10].

While not performed on our model, future trainers can optionally adjust the length of the nerve and vascular bundle to mask their location. Using different materials such as a Penrose drain, latex tubing, or ventilator tubing would allow learners to repeatedly puncture the vessels and also utilize this model as a vascular access trainer. Finally, different types of meat can be considered for the phantom, such as pork belly with a thick rind. This may better represent skin and subcutaneous tissue; however, it may significantly alter the cost of creating the model. 

## Conclusions

Ultrasound-guided foreign body identification is an increasingly essential skill for the Emergency Physician that requires practice and familiarity to effectively identify objects. Regional anesthesia is another skill that requires practice with realistic models to create procedural confidence and accuracy. Given that professional models can be prohibitively expensive, this paper outlines a simple approach to creating a quick, cheap, and realistic model. Other models have been created to provide learners with ample opportunity to develop these ultrasound skills; however, none have been designed with multiple modalities as described. 

We believe our chuck roast phantom accurately replicates human tissue and pathology and is ideal for learners to practice several skill sets at once. The model detailed in this article is practical, economical, simple to execute, and will be an effective training aid for learners.
